# Clinical efficacy and safety of posterior minimally invasive surgery in cervical spondylosis: a systematic review

**DOI:** 10.1186/s13018-022-03274-3

**Published:** 2022-08-13

**Authors:** Junqiao Lv, Jun Mei, Xiaoning Feng, Xuefeng Tian, Lin Sun

**Affiliations:** Third Hospital of Shanxi Medical University, Shanxi Bethune Hospital, Shanxi Academy of Medical Sciences, Tongji Shanxi Hospital, Taiyuan, 030032 China

**Keywords:** Cervical spondylosis, Posterior minimally invasive surgery, Systematic review, Percutaneous endoscopy, Channel, Keyhole

## Abstract

**Background:**

Posterior minimally invasive surgery has been increasingly used in in recent years for the clinical treatment of cervical spondylosis. However, this treatment remains challenging and has not been comprehensively reported. The aim of this study was to provide a systematic review of posterior minimally invasive treatment for cervical spondylosis to demonstrate the clinical efficacy and safety of this procedure.

**Method:**

We collected information from patients with myelopathy or radiculopathy cervical spondylosis who underwent posterior minimally invasive surgery and verified the clinical efficacy and safety of these surgeries with different measurement indicators from five electronic databases: the Nurick, visual analog scale score, Japanese Orthopaedic Association (JOA) score, Neck Disability Index (NDI), EuroQol Five Dimensions Questionnaire (EQ-5D) score, Short-Form Health Survey Physical Component Summary (SF12-PCS) questionnaire score, Short-Form Health Survey Mental Component Summary (SF12-MCS) questionnaire score, and the MOS item short form health survey (HF-36) score. The decompression effect, cervical spine stability, average surgery time, surgical blood loss volume, length of hospital stay, and related complications were included in the descriptive analysis. Reporting of this protocol followed the Preferred Reporting Items for Systematic Review and Meta-Analyses guidelines checklist.

**Results:**

We identified 14 observational studies of cervical spondylosis with 479 patients, mainly including 197 cases of myelopathy and 207 cases of radiculopathy. Channel and endoscopic techniques were used. This study was certified by PROSPERO: CRD42021290074. Significant improvements in the quantitative indicators (Neck-VAS in 9 studies, JOA in 7 studies, NDIs in 5 studies, Nurick, ARM-VAS, and EQ-5D in 2 studies each, and the SF12-PCS, SF12-MCS, and HF-36 in 1 study each) were observed between pre- and postoperation (*P* < 0.05), and satisfactory clinical significance was acquired in the descriptive indicators [average surgery time (94.56 ± 37.26 min), blood loss volume (68.78 ± 103.31 ml), average length of stay (2.39 ± 1.20 d), and cervical spine stability after surgery]. Additionally, we showed that there was a 4.9% postoperative complication rate and the types of complications that may occur.

**Conclusion:**

Posterior minimally invasive surgery is an effective and safe method for the treatment of cervical spondylosis and is a recommended optional surgical procedure for single-segment myelopathy and radiculopathy.

## Introduction

Degenerative cervical spondylosis (DCS) can be divided into a number of subdiseases, including cervical spondylotic radiculopathy (CSR) and cervical spondylotic myelopathy (CSM) [1–3]. CSR is a clinically common disease accounting for 60–70% of all types of DCS, and only a small number of patients who fail conservative treatment require surgical treatment, usually with anterior cervical surgery [4–6]. Regarding the treatment of CSM, anterior cervical surgery has been widely accepted for some patients [7, 8]. Nevertheless, with the increasing application of these surgeries, deficiencies and complications related to these procedures have been reported [9].

Recently, minimally invasive surgeries have been developed in spine surgery and have shown the same clinical effects as traditional open surgery for certain degenerative diseases in the lumbar spine [10, 11]. Minimally invasive surgery has certain advantages, such as less surgical trauma, lower blood loss volume, shorter length of stay, and faster postoperative recovery [12, 13]. In the treatment of DCS, some currently minimally invasive surgical methods, such as endoscopy techniques, channel techniques, keyhole techniques, and minimally invasive laminectomy, have been reported [1]. The effects and complications of these minimally invasive surgical procedures for DCS have been increasingly reported in recent years, so further systematic analysis of these reports is needed to facilitate a comprehensive evaluation for clinical reference regarding efficacy and safety [1, 14, 15].

By analyzing previous research, we argue in favor of the clinical efficacy of posterior minimally invasive surgery in DCS, which includes endoscopy technology, channel technology keyhole surgery, minimally invasive laminectomy, and so on. Many clinical indicators and whether these procedures can help patients avoid certain complications related to the traditional open surgical approach are further explored in this paper.

## Materials and methods

### Inclusion and exclusion criteria

This protocol is reported following the Preferred Reporting Items for Systematic Review and Meta-Analyses (PRISMA) guidelines checklist and is certified by PROSPERO: CRD42021290074.

#### Study type

Articles were excluded if they (1.) had a sample size of less than 10 consecutive patients or (2.) were an editorial, conference abstract, review, comment, letter, or animal study. The language was restricted to English.

#### Study population

The main patient inclusion criterion was CSR or CSM at a single level or multiple levels (documented using magnetic resonance imaging (MRI) and microcomputed tomography). Any patients with instability, significant anterior pathology, cervical spondylosis secondary to a tumor, trauma, severe ossification of the posterior longitudinal ligament, rheumatoid arthritis, septic spondylitis, and destructive spondylosis were excluded from the study.

#### Intervention

The intervention analyzed was posterior minimally invasive surgery for patients with different types of DCS. These include endoscopy technology, channel technology, and keyhole technology. The indication for posterior minimally invasive surgery was based on the current guidelines.

#### Outcome indicators

We used the Nurick score, visual analog scale (VAS), JOA (Japanese Orthopaedic Association (JOA) score, Neck Disability Index (NDI), EuroQol Five Dimensions Questionnaire (EQ-5D) score, Short-Form Health Survey Physical Component Summary questionnaire (SF-12 PCS) score, Short-Form Health Survey Mental Component Summary questionnaire (SF-12 MCS) score, and the MOS item short from health survey (HF-36) score as quantitative indicators. The decompression effect, cervical spine stability, average surgery time, surgical blood loss volume, length of hospital stay, and complications were also evaluated for a descriptive analysis.

#### Search strategy

Four electronic databases were searched for potential eligible studies: The Cochrane Library, PubMed, EMBASE, and Web of Science. Additionally, we manually searched the references from ClinicalTrials.gov and the gray literature. The search for the relevant literature was conducted from database inception until November 2021. We performed a literature search with the keywords “posterior approach,” “cervical spondylosis” and “minimally invasive surgery.” The full list used in the search strategy for PubMed was as follows: ((((((((Lumbarsacral Spondylosis) OR (Spondylosis, Lumbarsacral)) OR (Thoracic Spondylosis)) OR (Spondylosis, Thoracic)) OR (Cervical Spondylosis)) OR (Spondylosis, Cervical)) OR (Spondylosis Deformans)) AND ((((((((((((((((((((((((((Surgical Procedures, Endoscopic) OR (Procedure, Endoscopic Surgical)) OR (Procedures, Endoscopic Surgical)) OR (Surgical Procedure, Endoscopic)) OR (Endoscopy, Surgical)) OR (Surgical Endoscopy)) OR (Endoscopic Surgical Procedure)) OR (Endoscopic Surgical Procedures)) OR (Surgical Procedure, Minimal)) OR (Surgical Procedures, Minimal)) OR (Surgical Procedures, Minimal Access)) OR (Surgical Procedures, Minimally Invasive)) OR (Procedures, Minimally Invasive Surgical)) OR (Minimal Surgical Procedure)) OR (Minimal Surgical Procedures)) OR (Minimally Invasive Surgery)) OR (Minimally Invasive Surgeries)) OR (Surgeries, Minimally Invasive)) OR (Surgery, Minimally Invasive)) OR (Procedure, Minimal Surgical)) OR (Procedures, Minimal Access Surgical)) OR (Procedures, Minimal Surgical)) OR (Minimally Invasive Surgical Procedure)) OR (Minimal Access Surgical Procedures)) OR (channel)) OR (tunnel))) AND (posterior approach). This strategy was also applied for the other databases searched.

### Literature screening and data extraction

Two researchers independently screened the literature and extracted and cross-checked the data; in cases of disagreement, they discussed and resolved the disagreement, or a third researcher was consulted to assist in the determination. For studies lacking information, the original authors were contacted. The extracted information included the following: (1) basic information on the included studies, including author names, year of publication, and country; (2) basic characteristics of the study population, including sample size and age; (3) specific details of the interventions; (4) key elements of the risk of bias evaluation; and (5) data on the outcome indicators of interest.

### Assessment of the risk of bias in the included studies

The risk of bias in the included studies was evaluated using the risk of bias evaluation tool recommended by the METHODOLOGICAL INDEX FOR NONRANDOMIZED STUDIES (MINORS): (1.) A clearly stated aim: The question addressed should be precise and relevant in light of the available literature. (2.) Inclusion of consecutive patients: All patients potentially fit for inclusion (satisfying the criteria for inclusion) should be included in the study during the study period (no exclusion or details about the reasons for exclusion). (3.) Prospective data collection: Data should be obtained according to a protocol established before the beginning of the study. (4.) Endpoints appropriate to the aim of the study: Unambiguous explanation of the criteria used to evaluate the main outcome should be in accordance with the question addressed by the study. Additionally, the endpoints should be assessed on an intention-to-treat basis. (5.) Unbiased assessment of the study endpoint: Blind evaluation of objective endpoints and double-blind evaluation of subjective endpoints should be performed. Otherwise, the reasons for not blinding should be stated. (6.) Follow-up period appropriate to the aim of the study: The follow-up should be sufficiently long to allow the assessment of the main endpoint and possible adverse events. (7.) Loss to follow-up less than 5%: All patients should be included in the follow-up. Otherwise, the proportion lost to follow-up should not exceed the proportion experiencing the major endpoint. (8.) Prospective calculation of the study size: Information on the size of the detectable difference in interest should be obtained, the 95% confidence interval should be calculated according to the expected incidence of the outcome event, and information about the level of statistical significance and estimates of power should be obtained when comparing the outcomes. The risk of bias evaluation was performed independently by 2 evaluators, and the results were cross-checked and discussed.

### Statistical analysis

If evidence of clinical homogeneity could be established, then the study was included in the meta-analysis. We performed a qualitative synthesis of findings from scientifically admissible studies to develop evidence statements according to the principles of best-evidence synthesis. GraphPad Prism 8.0 was used for statistical analysis and graphing.

## Results

### Flowchart of the literature screening process and results

**Fig. 1:**
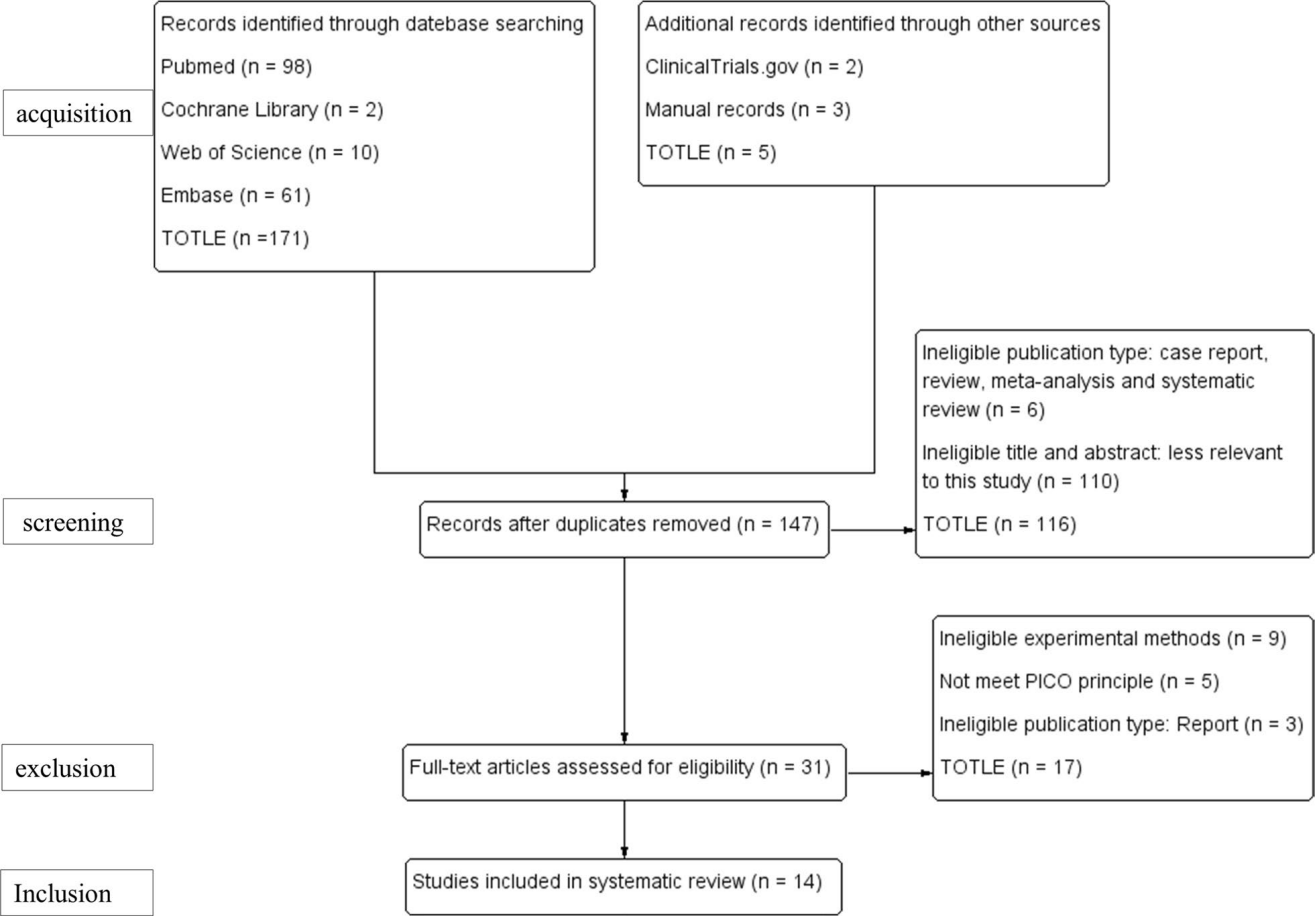
Flowchart of the literature screening process and results

A PRISMA diagram was used to report the search results and screening process. We identified a total of 176 studies: 98 studies in PubMed, 10 studies in Web of Science, 61 studies in Embase, 2 studies in the Cochrane Library, 2 studies in ClinicalTrials.gov, and 3 studies from the manual search. After the initial screening, a total of 147 studies remained (removal of duplicate records). After reviewing the titles and abstracts of the 147 studies, 31 studies remained (removal of ineligible publications: case reports, reviews, meta-analyses, and systematic reviews and studies with ineligible titles and abstracts, which were less relevant to this study). Finally, we assessed the full text, and a consensus was reached regarding the inclusion of 14 articles (13 case series studies and 1 cohort study) (Fig. 1).

### Basic characteristics of the included studies

**Fig. 2:**
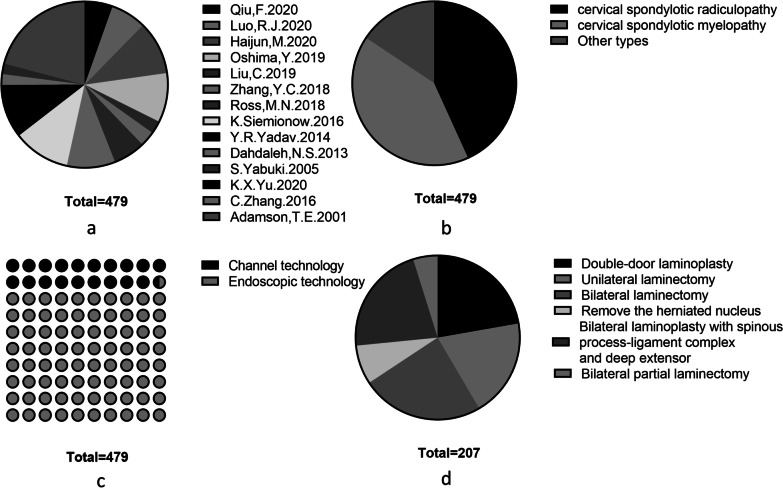
**a**. Proportion of patients in each study. **b**. Proportion of patients with radiculopathy, myelopathy and other subtypes. **c**. Proportion of patients who underwent the channel and endoscopic techniques. **d**. Proportion of patients with myelopathy who underwent different surgical methods

Fourteen articles involving 479 patients who underwent DCS were included. We extracted information related to the study characteristics, including the country of origin of the study population, study type, follow-up time, type of cervical spondylosis, study sample size, sex of the participants, average participant age, and surgical procedures of treatment. The characteristics of the 14 articles are presented in Table 1, and the basic characteristics of the included literature are summarized by category (Table 2, Fig. 2).

**Table 1:** Characteristic of included studies

Study	Country	Study type	Follow-up time (months)	Disease	Sample size	Sex (male:female)	Age	Interventions	Populations
Yu [16]	Japan	Single-arm study	12	Single-segment myelopathy	16	6:10	52	Posterior percutaneous endoscopic cervical discectomy	Single-segment myelopathy
Qiu [17]	China	Single-arm study	9	Cervical spondylotic radiculopathy	10	6:4	41.5	Posterior intervertebral foraminal discectomy via Delta channel	Cervical spondylotic radiculopathy
Luo [18]	China	Single-arm study	25.7	Cervical spondylotic radiculopathy	33	16:17	51.3	Keyhole foraminotomy via a percutaneous posterior full-endoscopic approach	Cervical spondylotic radiculopathy
Haijun [19]	China	Single-arm study	24.36	Cervical spondylotic radiculopathy	50	23:27	61.19	Posterior percutaneous endoscopic cervical discectomy	Cervical spondylotic radiculopathy
Oshima [20]	Japan	Double-arm study	27.8	Cervical spondylotic myelopathy	46	32:14	63.4	Cervical microendoscopic interlaminar decompression	Cervical spondylotic myelopathy
Liu [13]	China	Single-arm study	6	Cervical intervertebral disk herniation	12	5:7	51.92	Posterior percutaneous endoscopic cervical discectomy	Cervical intervertebral disk herniation
Zhang [21]	China	Single-arm study	9	Cervical spondylotic radiculopathy	14	6:8	40.5	Percutaneous transforaminal endoscopic discectomy via posterior approach	Cervical spondylotic radiculopathy
Ross [22]	Portland	Single-arm study	27	Cervical spondylotic myelopathy	30	30:0	69	Minimally invasive cervical foraminotomy (channel)	Cervical spondylotic myelopathy
Zhang [23]	China	Single-arm study	28	Cervical spondylotic myelopathy	45	25:20	58	Cervical endoscopic laminoplasty	Cervical spondylotic myelopathy
Siemionow [24]	United States	Single-arm study	24	Single- or multilevel cervical spondylosis	53	18:35	53	Indirect decompression and posterior cervical fusion using a cervical intervertebral cage (channel)	Single- or multilevel cervical spondylosis
Yadav [25]	India	Single-arm study	19	Multilevel cervical compressive myelopathy	50	38:12	55.8	Endoscopic decompression of cervical spondylotic myelopathy	Multilevel cervical compressive myelopathy
Dahdaleh [14]	United States	Single-arm study	20.3	Cervical spondylotic myelopathy	10	8:2	67	Minimally invasive endoscopically assisted decompression of stenosis	Cervical spondylotic myelopathy
Yabuki [26]	Japan	Single-arm study	14.9	Cervical spondylotic myelopathy	10	5:5	66	Endoscopic partial laminectomy	Cervical spondylotic myelopathy
Adamson [32]	United States	Single-arm study	14.8	Unilateral radiculopathy	100	63:37	46.6	Microendoscopic posterior cervical laminoforaminotomy	Unilateral radiculopathy

**Table 2:** Summary characteristics of included studies

Characteristics	Values
Total no. of trials (no. of participants)	14 (479)
Median follow-up (years)	20.7 (6–27.8)
Follow-up at least one year	11 (443)
Median no. of participants	34 (10–100)
Proportion female, %	41.3
Median age (years)	55.1
*Country*	
European	1 (30)
American	3 (163)
Asian-Pacific	10 (286)

### Methodological quality of the included studies

We assessed the risk of bias using the MINORS (Table 3).

**Table 3:** Methodological items for nonrandomized studies

Study	A clearly stated aim	Inclusion of consecutive patients	Prospective collection of data	Endpoints appropriate to the aim of the study	Unbiased assessment of the study endpoint	Follow-up period appropriate to the aim of the study	Loss to follow-up less than 5%	Prospective calculation of the study size
Yu [16]	2	2	2	2	0	1	2	0
Qiu [17]	2	2	2	2	0	1	2	0
Luo [18]	2	2	2	2	0	2	2	0
Haijun [19]	2	2	2	2	0	2	2	0
Oshima [20]	2	2	2	2	0	2	2	0
Liu [13]	2	2	2	2	0	1	2	0
Zhang [21]	2	2	2	2	0	1	2	0
Ross [22]	2	2	1	2	0	2	2	2
Zhang [23]	2	2	2	2	0	2	2	0
Siemionow [24]	2	2	2	2	0	2	1	0
Yadav [25]	2	2	2	2	0	2	2	0
Dahdaleh [14]	2	2	2	2	0	2	2	0
Yabuki [26]	2	2	2	2	0	1	2	0
Adamson [32]	2	2	0	0	0	2	2	0

The items are scored 0 (not reported), 1 (reported but inadequate) or 2 (reported and adequate)

### Systematic review

Among the posterior minimally invasive surgical approaches for DCS, a total of 479 patients across the 14 studies achieved spinal nerve root or spinal cord decompression after different surgical approaches were performed (channel or endoscopy), and no patients experienced postoperative cervical instability. Eleven studies reported average surgery times ranging from 44 to 163.6 min (94.56 ± 37.26 min). Nine studies reported blood loss volumes ranging from 18 to 360 ml (68.78 ± 103.31 ml), and 5 of the studies did not clearly indicate the amount of blood loss. In addition, only 6 studies reported the average length of hospital stay; the longest average length of stay was 4.66 days, and the shortest average length of stay was 1.1 days (2.39 ± 1.20 days). A total of 12 studies recorded postoperative complications, with 92 patients in five studies experiencing no complications. In the remaining 7 studies, 18 patients experienced complications; the most common was dural tears (4 patients), the least common was disk herniation recurrence (1 patient) and a loose screw (1 patient); other reported complications included bleeding (3 patients), decreased muscle strength (3 patients), dysesthesia (2 patients), pain and numbness (2 patients), and root injury (2 patients). Finally, only 6 studies reported MacNab criteria, and the proportion of patients with a poor outcome was less than 10% (Table 4).

**Table 4:** Quantitative analysis of endings

Study	Decompression	Postoperative cervical instability	Average surgery time (minutes)	Blood loss (ml)	Stay in hospital (days)	Complications	MacNab
Yu [16]	YES	NO	44	Not mentioned	Not mentioned	NO	Excellent: 8 (50%) Good: 6 (37.5%) Fair: 2 (12.5%)
Qiu [17]	YES	NO	90	40	Not mentioned	NO	Not mentioned
Luo [18]	YES	NO	62	Not mentioned	1.6	1 case in dural tears 1 case in transient postoperative dysesthesia	Excellent: 19(57.6%) Good: 13(39.4%) Fair: 1(3.0%)
Haijun [19]	YES	NO	75.46	20.33	4.66	1 case in aggravated pain and numbness 2 cases in dural tear 1 case in decreased muscle strength 1 case in disk herniation recurrence	Excellent: 35(70.0%) Good: 12(24.0%) Acceptable: 2(4.0%) Poor: 1(2.0%)
Oshima [20]	YES	NO	Not mentioned	18	Not mentioned	NO	Not mentioned
Liu [13]	YES	NO	74.92	Not mentioned	3.17	1 case in hypoaesthesia	Not mentioned
Zhang [21]	YES	NO	75	40	Not mentioned	Not mentioned	Not mentioned
Ross [22]	YES	NO	142	Not mentioned	Not mentioned	1 case in no motor function in the right hand intrinsic muscles	Not mentioned
Zhang [23]	YES	NO	119	360	Not mentioned	1 case upper limb motion dysfunction 1case mini-screw was loosened	Excellent: 13(28.8%) Good: 25(55.6%) Fair: 7(15.6%)
Siemionow [24]	YES	NO	59.15	32.83	Not mentioned	1 case in right shoulder pain	Not mentioned
Yadav [25]	YES	NO	135	30	2.2	1 case in minor dural tears 3 case in minor bleedings from muscles 2 case in c5 root injury	Not mentioned
Dahdaleh [14]	YES	NO	Not mentioned	32.3	1.6	NO	Excellent: 4 (40.0%) Good: 3(30.0%) Fair: 2(20.0%) Poor: 1(10.0%)
Yabuki [26]	YES	NO	163.6	45.5	Not mentioned	NO	Not mentioned
Adamson [32]	YES	NO	Not mentioned	Not mentioned	1.1	Not mentioned	Excellent: 91(91.0%) Good: 6(6.0%) Fair: 3(3.0%)

**Fig. 3:**
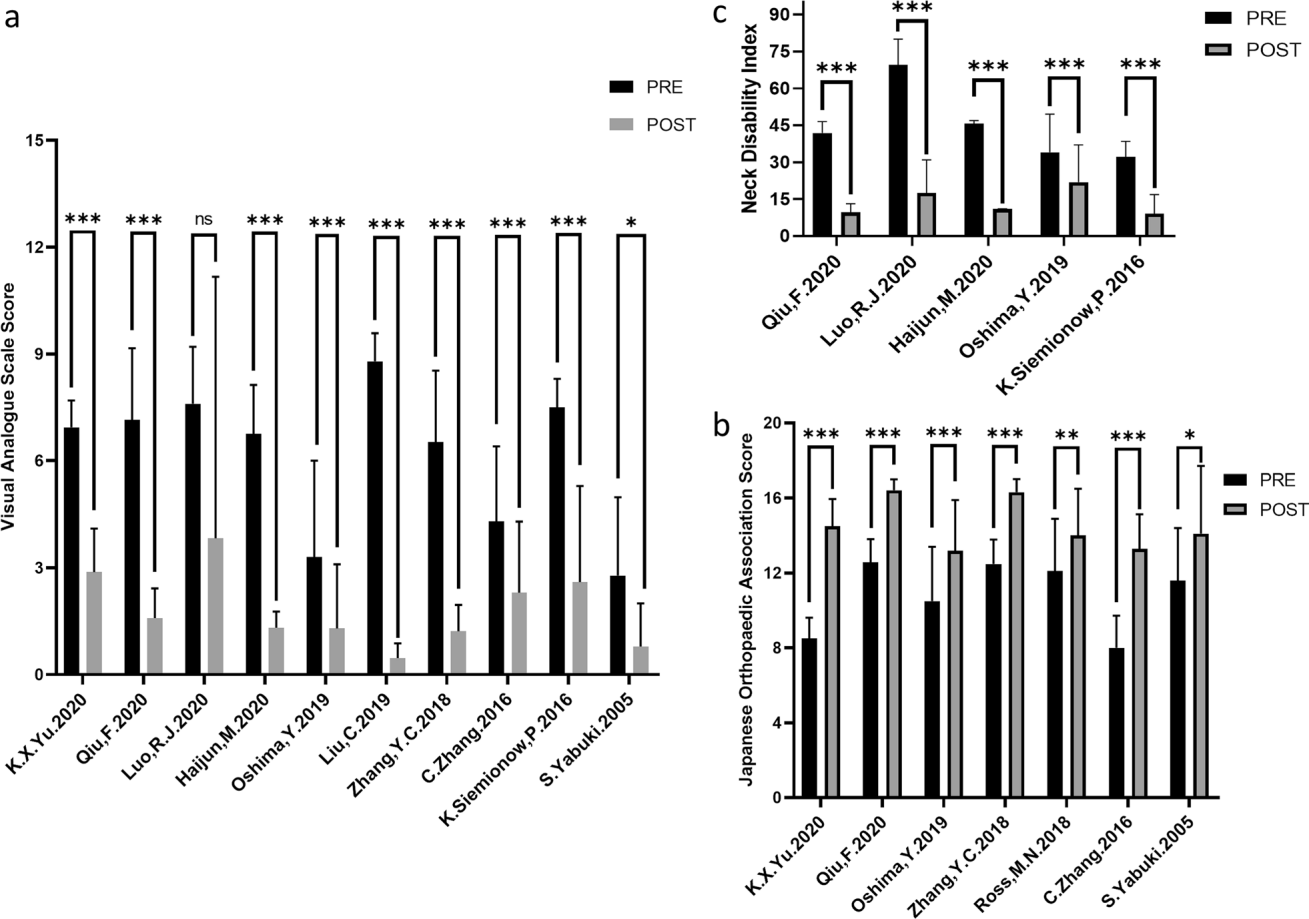
**a**. Pre- and postoperative indicators for visual analog scale score (Neck). **b**. Pre- and postoperative indicators for the Japanese Orthopedic Association score. **c**. Pre- and postoperative indicators for the Neck Disability Index

The patients’ Nurick scores were reported in 2 studies [14, 25]. There were significant differences in the pre- and postoperative study data reported by Yadav [25] (*P* < 0.05) and Dahdaleh [14] (*p* < 0.05) (Table 5). Patient Neck-VAS scores were reported in 10 studies [13, 16–21, 23, 24, 26]. There were significant differences in the pre- and postoperative study data, and one outlier was reported (Luo [18] (*P* > 0.05), Qiu [17] (*P* < 0.001), Yu [16] (*P* < 0.001), Zhang [23] (*p* < 0.001), Yabuki [26] (*p* < 0.05), Haijun [19] (*P* < 0.001), Oshima, S [20] (*P* < 0.001), Liu [13] (*p* < 0.001), Zhang [21] (*p* < 0.001), and Siemionow [24] (*p* < 0.001)) (Table 5, Fig. 3). The JOA scores were reported in 7 studies [16, 17, 20–23, 26], and significant differences in the pre- and postoperative study data were found (Yu [16] (*p* < 0.001), Zhang [23] (*p* < 0.001), Yabuki [26] (*p* < 0.05), Qiu [17] (*P* < 0.001), Oshima [20] (*P* < 0.001), Zhang [21] (*P* < 0.001), and Ross [22] (*P* < 0.01)) (Table 5, Fig. 3). The NDIs were reported in 5 studies [13, 17, 19, 20, 24], and there were significant differences in the pre- and postoperation study data (Qiu [17] (*P* < 0.001), Luo [13] (*P* < 0.001), Haijun [19] (*P* < 0.001), Oshima, S [20] (*P* < 0.001), and Siemionow [24] (*p* < 0.001)) (Table 5, Fig. 3). Patient ARM-VAS scores were reported in 2 studies [19, 24], and significant differences were observed in the pre- and postoperative study data (Haijun [19] (*P* < 0.01) and Siemionow [24] (*p* < 0.0001)) (Table 5). Patient EQ-5D scores were reported in 2 studies [19, 20], with significant differences found in the pre- and postoperation study data by both Haijun [19] (*P* < 0.01) and Oshima [20] (*P* = 0.05) (Table 5). Patient SF12-PCS scores were reported in 1 study [24], and significant differences were found in the pre- and postoperation study data (Siemionow [24] (*p* < 0.0001)). Patient SF12-MCS scores were reported in 1 study [24], and significant differences were observed in the pre- and postoperation study data (Siemionow, [24] (*p* < 0.0001)). Patient HF-36 scores were reported in 1 study [21], and significant differences found in the pre- and postoperation study data (Zhang [21]) (Table 5) (*P* < 0.05).

**Table 5:** Quantitative analysis of endings

Study	Nurick		Neck-VAS		JOA		NDI		Arm—VAS score		EQ-5D score		SF-12PCS		SF-12MCS		HF-36	
	Pre	Post	Pre	Post	Pre	Post	Pre	Post	Pre	Post	Pre	Post	Pre	Post	Pre	Post	Pre	Post
Yu [16]			6.94 ± 0.75	2.88 ± 1.22	8.50 ± 1.12	14.50 ± 1.46												
Qiu [17]			7.15 ± 2.01	1.59 ± 0.83	12.57 ± 1.24	16.42 ± 0.58	41.82 ± 4.71	9.59 ± 3.52										
Luo [18]			7.6 ± 1.6	3.83 ± 7.34			69.5 ± 10.5	17.54 ± 13.40										
Haijun [19]			6.75 ± 1.38	1.32 ± 0.45			45.67 ± 1.20	10.93 ± 0.11	8.70 ± 0.13	1.88 ± 0.26	0.36 ± 0.6	0.71 ± 0.17						
Oshima [20]			3.3 ± 2.7	1.3 ± 1.8	10.5 ± 2.9	13.2 ± 2.7	34.0 ± 15.5	21.9 ± 15.1			0.58 ± 0.11	0.73 ± 0.17						
Liu [13]			87.91 ± 7.88	4.67 ± 4.10														
Zhang [21]			6.52 ± 2.01	1.22 ± 0.74	12.48 ± 1.31	16.32 ± 0.69											61.5 ± 14.2	79.2 ± 16.5
Ross [22]					12.1 ± 2.8	14 ± 2.5												
Zhang [23]			4.3 ± 2.1	2.3 ± 2.0	8.0 ± 1.72	13.29 ± 1.85												
Siemionow [24]			7.5 ± 0.8	2.6 ± 2.7			32.2 ± 6.2	9.1 ± 7.7	7.4 ± 0.9	2.6 ± 2.9			34.3 ± 6.0	43.7 ± 8.4	40.3 ± 7.6	51.4 ± 8.8		
Yadav [25]	2.6 ± 0.67	0.92 ± 0.37																
Dahdaleh [14]	1.6 ± 0.7	0.3 ± 0.7																
Yabuki [26]			2.78 ± 2.23	0.79 ± 1.21	11.6 ± 2.8	14.1 ± 3.6												
Adamson [32]

**Fig. 4:**
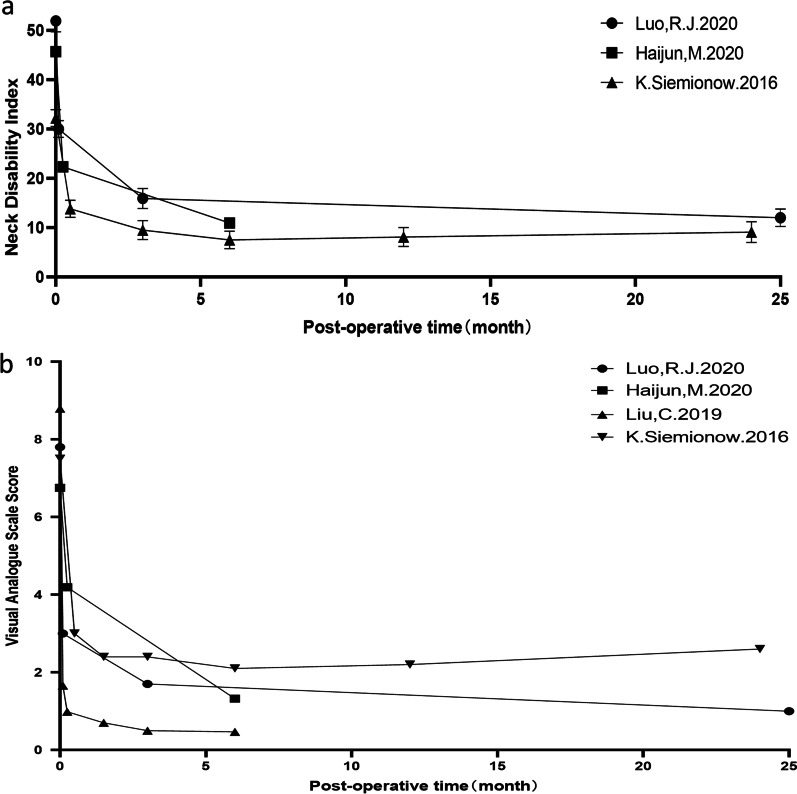
**a**. Postoperative Neck Disability Index versus time. **b**. Postoperative visual analog scale score (Neck) versus time

The reported changes in the NDI and Neck-VAS indicators over time with posterior minimally invasive surgery showed that these patients achieved better long-term results in terms of pain relief and disability reduction, but some outliers remained (NDI and Neck-VAS scores of K. Siemonow at 24 months) (Fig. 4).

**Fig. 5:**
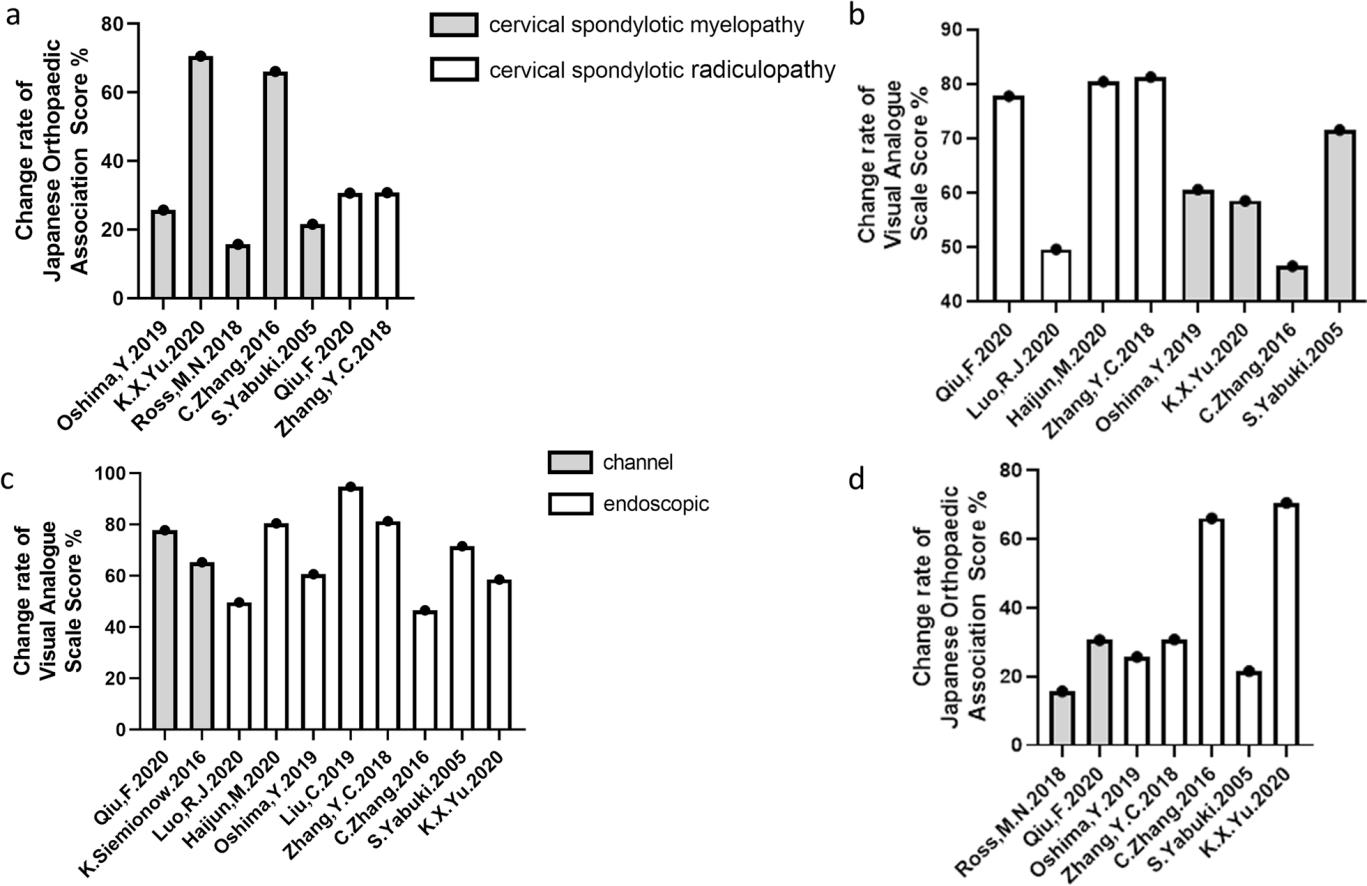
**a**. Change rate of JOA score between myelopathy and radiculopathy. **b**. Change rate of VAS score (Neck) between myelopathy and radiculopathy. **c**. Change rate of JOA score between channel and endoscopic technique. **d**. Change rate of VAS score (Neck) between channel and endoscopic technique

Both surgical methods and types of cervical spondylosis had good results in terms of qualitative outcome indicators (decompression effect, cervical instability). However, we found that by comparing the quantitative indicators of posterior minimally invasive surgery and two types of cervical spondylosis, low-quality evidence showed the differences in the channel and endoscopy techniques between the pre- and postoperation study data. At the same time, among these indicators, all of the indicators for radiculopathy and most of the indicators for myelopathy showed differences, except for VAS (Luo, R. J. 2020) (Table 6, Fig. 5).

**Table 6:** Subgroup characteristics of included studies

Subgroup title	No. of trials	No. of participants	Decompression	Cervical instability	Significant difference*
Overall	14	479	YES	YES	PARTIAL**
*Population characteristics*					
Cervical spondylotic radiculopathy	5	207	YES	NO	PARTIAL**
Cervical spondylotic myelopathy	7	197	YES	NO	YES
Other types	2	75	YES	NO	YES
*Surgical approach*					
Channel technique	3	93	YES	NO	YES
Endoscopic technique	11	386	YES	NO	YES

*Indicates significant difference in the changes of quantitative outcome indicators pre- and postsurgery

**Indicates significant differences in changes in most quantitative outcome indicators before and after surgery

**Fig. 6:**
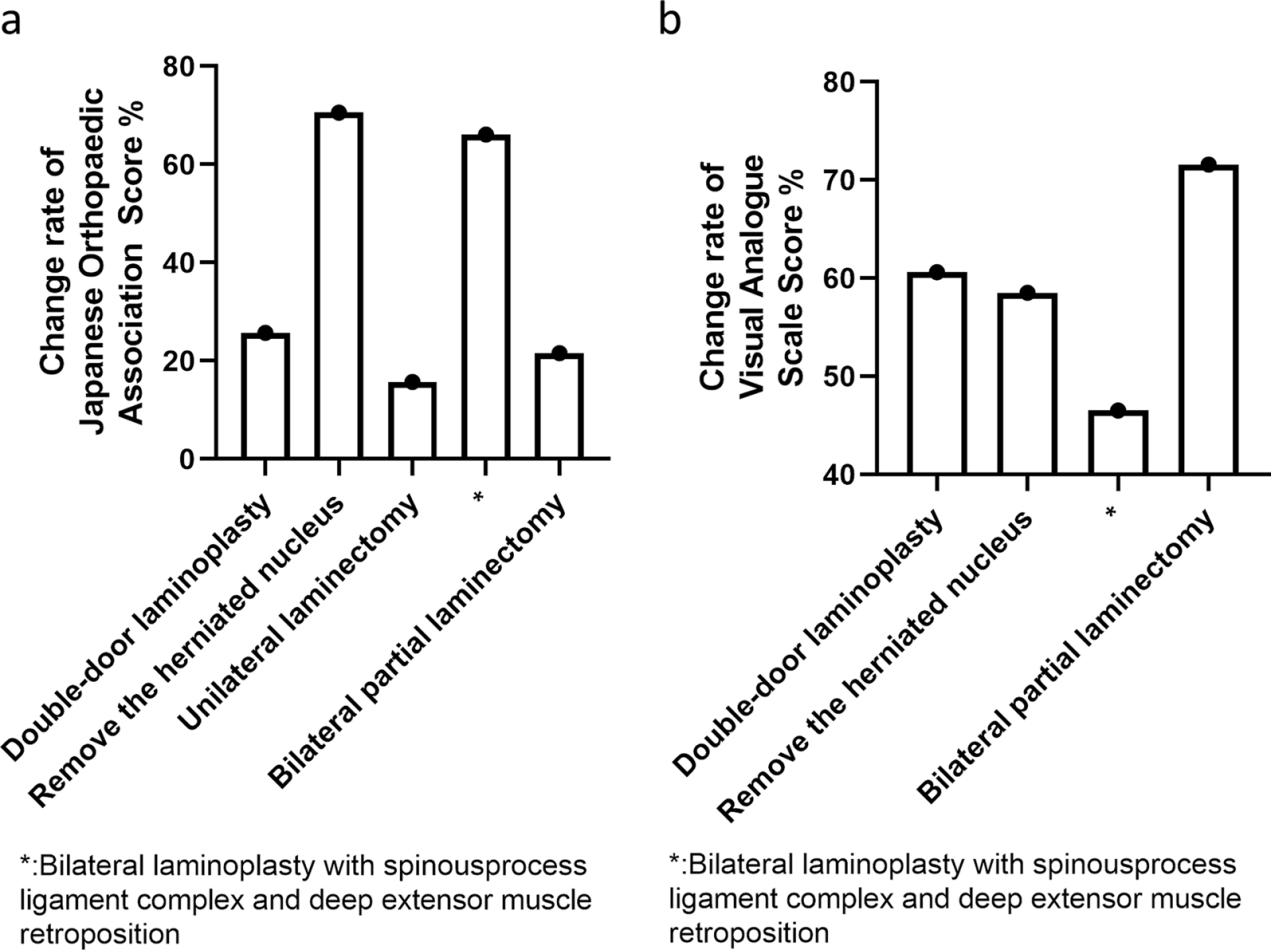
**a**. Change rate of JOA score for different types of surgery. **b**. Change rate of VAS score (Neck) for different types of surgery

More importantly, we compared the quantitative indicators of patients with CSM treated with different surgical approaches. There was lower quality evidence showing that the most significant surgical approach for improving the change rate of JOA was removal of the herniated nucleus, and the least significant was unilateral laminectomy. Similarly, the most significant surgical approach for reducing the change rate of Neck-VAS was bilateral partial laminectomy, and the least significant was bilateral laminoplasty with spinous process ligament complex and deep extensor muscle retroposition (Table 7, Fig. 6).

**Table 7:** Summary characteristics of included studies (cervical spondylotic myelopathy)

Characteristics	No. of trials (no. of participants)	Study (no. of participants)
Double-door laminoplasty	1 (46)	Oshima [20] (46)
Unilateral laminectomy	2 (40)	Dahdaleh [14] (10), Ross [22] (30)
Bilateral laminectomy	1 (50)	Yadav [25] (50)
Herniated nucleus removal	1 (16)	Yu [14] (16)
Bilateral laminoplasty with spinous process ligament complex and deep extensor muscle retroposition	1 (45)	Zhang [23] (45)
Bilateral partial laminectomy	1 (10)	Yabuki [26] (10)

## Discussion

This systematic review mainly focuses on the clinical efficacy and safety of posterior minimally invasive surgery for DCS. Fourteen or articles were included in the study, including 13 case series studies and 1 cohort study. In our review, patients with CSM and CSR accounted for 85% of the total number of patients, and the number of patients with the two types (CSM and CSR) was similar. Posterior minimally invasive surgery is widely used in single-segment DCS, especially in CSR, and in recent years, researchers have paid more attention to the role of different posterior minimally invasive procedures in CSM, which is also reflected in our study. From the results of the analysis, it can be concluded that there were significant differences in the data on pain, function and quality of life after posterior minimally invasive approaches when compared with those before surgery. At the same time, some results showed that posterior minimally invasive surgery in DCS had some long-term effects on reducing pain and improving local disability. Interestingly, average surgery time, surgical blood loss volume, and length of hospital stay were also satisfactory. Postoperative complications are a paramount concern, with an approximately 4.9% complication rate reported after posterior minimally invasive approaches among the 365 patients.

There are limitations to the conclusions drawn from the current body of evidence, as none of the studies were RCTs. Additionally, the sample sizes are too small and may be difficult to follow-up in surgical cases. Although we classified the studies in terms of the different posterior minimally invasive procedures, each surgical team had subtle differences in surgical style, which may have increased the heterogeneity of the study. Our findings are at risk of publication bias: R. J. Luo et al. reported outliers on the Neck-VAS, for example, while K. Siemionow reported the NDI and Neck-VAS at 24 months. This may be because surgeons are reluctant to publish studies that adversely affect surgical outcomes.

Surgical intervention is required for patients with DCS to relieve symptoms after failing to respond to systematic conservative treatment. For CSR patients, pain is the most significant factor affecting quality of life, and the most significant benefit of posterior minimally invasive approaches is the reduction in pain radiating to the neck and upper limbs [27]. For severe patients with decreased muscle stiffness of the upper extremities and gait disturbances, posterior minimally invasive approaches are more effective. However, there are advantages and disadvantages to each surgical procedure. It is necessary to understand the indications of each and choose the correct procedure for each patient to achieve the best outcomes and minimize the risk of complications, which include (1) foraminal disk herniation (mainly unilateral upper limb pain); (2) single- or multisegmental foraminal stenosis (unilateral upper limb pain); and (3) persistent symptoms despite a history of anterior cervical discectomy and fusion. Contraindications include (1) axial neck pain; (2) cervical instability; and (3) the presence of cervical goose neck deformity.

The posterior minimally invasive posterior surgery in DCS reduces the impact on the homeostasis of the internal environment and reduces the patient's own recovery burden, making the procedure more acceptable to patients. At the same time, this surgical approach causes less damage to the soft tissues at the back of the neck, requiring less soft tissue to be stripped away and greatly reducing postoperative axial symptoms [28]. Interestingly, it does not require immobilization, reduces the impact of surgical access on the stability of the cervical spine and maintains the mobility of the cervical segments [29]. It also does not require additional blood transfusion and has a short and inexpensive hospital stay. Additionally, surgical intervention through posterior minimally invasive surgery may reduce the need for fluoroscopy [30]. However, the smaller surgical space results in a limited decompression area, and it is difficult to achieve reconstruction of the cervical sagittal sequence and stability. Finally, complex posterior minimally invasive surgical techniques result in a steeper learning curve. Future research should focus on the effects, long-term prognosis and risk of recurrence of different posterior minimally invasive surgeries for DCS, especially the more complex CSM. It is also necessary to implement such studies in a large group of patients with a comparative cohort. Our study reported that these surgical approaches did not result in changes in the biomechanical structure of the cervical spine, but there is a lack of specific data to support this view. There are still concerns regarding problems related to accelerated postoperative degeneration after facet resection. There is an urgent need to establish a uniform and standardized nomenclature and procedure for posterior minimally invasive surgery to assist teaching surgeons and improving their skills [31].

## Conclusion

Posterior minimally invasive surgery is an effective and safe method for the treatment of cervical spondylosis myelopathy and radiculopathy patients, especially simple, single-segment cervical spondylosis.

## Data Availability

The datasets used or analyzed during the current study are available from the corresponding author on reasonable request.
